# Comparison of Cell-Free Extracts from Three Newly Identified *Lactobacillus plantarum* Strains on the Inhibitory Effect of Adipogenic Differentiation and Insulin Resistance in 3T3-L1 Adipocytes

**DOI:** 10.1155/2021/6676502

**Published:** 2021-04-15

**Authors:** Naeun Oh, Jaehoon Lee, Hyewon Kim, Mijin Kwon, Jeongmin Seo, Sangho Roh

**Affiliations:** ^1^Cellular Reprogramming and Embryo Biotechnology Laboratory, Dental Research Institute, Seoul National University School of Dentistry, Seoul 08826, Republic of Korea; ^2^Biomedical Research Institute, NeoRegen Biotech Co., Ltd., Gyeonggi-do 16614, Republic of Korea

## Abstract

Obesity and associated metabolic disorders, including cardiovascular disease and diabetes, are rapidly becoming serious global health problems. It has been reported that *Lactobacillus plantarum* (*L. plantarum*) extracts have the beneficial activities of antiobesity and antidiabetes, although few studies have compared the beneficial effects among various *L. plantarum* extracts. In this study, three new *L. plantarum* (named LP, LS, and L14) strains were identified, and the antiobesogenic and diabetic effects of their extracts were investigated and compared using 3T3-L1 cells *in vitro*. Lipid accumulation in maturing 3T3-L1 cells was significantly decreased by the addition of LS and L14 extracts. The mRNA expression levels of *Pparγ*, *C/ebpα*, *Fabp4*, *Fas*, and *Dgat1* were significantly decreased by the addition of LP, LS, and L14 extracts. Interestingly, the protein expression levels of PPAR*γ*, C/EBP*α*, FABP4, and FAS were downregulated in mature 3T3-L1 cells with the addition of the L14 extract. Moreover, the LS and L14 extract treatments stimulated glucose uptake in maturing adipocytes. The L14 extract treatments exhibited a significant reduction in TNF-*α* protein expression, which is a key factor of insulin resistance in adipocytes. Of the three extracts, L14 extract markedly reduced adipogenic differentiation and insulin resistance *in vitro*, suggesting that the L14 extract may be used as a therapeutic agent for obesity-associated metabolic disorders.

## 1. Introduction

Overweight and obesity are deemed as serious public health problems [1, 2]. Excess bodyweight causes hyperlipidemia, hypertension, and cancer and accounts for a considerable proportion of morbidity and mortality, with nearly 300,000 deaths reported annually [3, 4]. Obesity-induced insulin resistance is one of the hallmarks of metabolic syndrome and eventually triggers type 2 diabetes and cardiovascular disease [5]. Insulin resistance has been defined as the decreased ability of insulin to stimulate glucose transport and metabolism in skeletal muscle and adipocytes [6]. Recently, several studies have been conducted using 3T3-L1 preadipocytes to investigate the antidiabetic potential as a therapeutic target [7–9]. Therefore, studies have concentrated on the discovery and development of new substances that have preventive and therapeutic effects on obesity and obesity-related diseases.

Lactic acid bacteria (LAB) have special physiological activities and are deemed as safe bacteria [10]. In previous studies, probiotics were demonstrated to have health-promoting effects on various diseases, including cancer [11], allergy symptoms [12], hypertension [13], and obesity [14]. In particular, *Lactobacillus* is the most commonly used LAB microorganism in probiotic products [10]. Moreover, the antiobesity and antidiabetic effects of *Lactobacillus* were reported [7, 15–17]. However, the use of live probiotics poses challenges in production and storage [18]. In addition, live microbes not only have the potential for infectivity or *in situ* toxin production when administrated but they also risk contamination when they are treated in cells [7, 19]. Since there are drawbacks to using live organisms, previous studies have been conducted using extracts obtained by culturing LAB in the medium and then lysing the bacteria [20]. To date, it has been shown that the *Lactobacillus* extract significantly inhibits adipogenic differentiation of 3T3-L1 cells [21]. Moreover, the cell extracts of *Lactobacillus plantarum* (*L. plantarum*) have been studied to evaluate their inhibitory effect on 3T3-L1 adipocytes [22, 23]. Thus, studying the *Lactobacillus* extract can be an effective approach to treating obesity.

*L. plantarum* can be divided into hundreds of subspecies, and new strains are still being discovered [24]. It has been shown that significant strain-to-strain variation was found in the proteome, genomic, and metabolic diversity among *L. plantarum* strains [25–27]. However, studies rarely reported the differences in the degree of effects among numerous *L. plantarum* stains, particularly antiobesity and antidiabetes. Therefore, this study focused on comparing the effects of newly identified *L. plantarum* extracts on adipogenic differentiation and obesity-induced insulin resistance in 3T3-L1 cells.

## 2. Materials and Methods

### 2.1. Isolation and Identification of *Lactobacillus* Strains

Three *L. plantarum* strains were newly identified. For this study, several strains that were preliminarily screened were obtained from NeoRegen Biotech (Suwon, Korea), including *L. plantarum* LS (LS), *L. plantarum* LP (LP), and *L. plantarum* L-14 (L14) strains. L14 (KCTC13497BP) was isolated from a traditional Korean rice-fermented food containing flatfish via a series of screening. These strains were cultured in a de Man, Rogosa, and Sharp (MRS; Hardy Diagnostics, Santa Maria, CA, USA) broth at 35°C. To identify these *Lactobacilli*, API 50 CHL strips (Microgiene, Gunpo, Korea) were used according to the manufacturer's recommendations. The results of their reactions were inputted into the APIWEB™ software (Biomerieux S.A., La Balme les Grottes, France), and each strain was identified.

### 2.2. Preparation of *Lactobacillus* Extracts

LS, LP, and L14 were cultured in the MRS broth at 35°C for 18 h for precultivation. Then, these were 1% inoculated for main-cultivation in the MRS broth and cultured at 35°C for 18 h. The three types of cultured *L. plantarum* strains were harvested by centrifugation (10,000 × *g* for 10 min at 4°C), respectively, and washed two times with phosphate-buffered saline (PBS). Then, these were washed with distilled water to remove the MRS broth and PBS completely. Then, each washed *L. plantarum* strain was sonicated (Sonics, Stratford, CT, USA) on ice for 30 min to break the cell wall of bacteria and to make the extracts homogeneous. To remove the cell debris, the extracts were centrifuged at 10,000 × *g* for 20 min at 4°C. The supernatants were passed through a 0.45 *μ*m filter and frozen at −80°C overnight and were then freeze-dried and reconstituted with PBS before use.

### 2.3. Cell Culture and Differentiation of Mouse 3T3-L1 Cells

3T3-L1 preadipocytes were provided by the American Type Culture Collection. The cells were grown in a culture medium containing Dulbecco's modified Eagle's medium (DMEM; WELGENE, Gyeongsan-si, Korea), 10% fetal bovine serum (FBS; Hyclone Laboratories Inc., Logan, UT, USA), and 1% penicillin/streptomycin (PS; Life Technologies, Camarillo, CA, USA). The cells were cultured at 37°C in an incubator containing a humidified atmosphere of 5% CO_2_. 3T3-L1 cells were seeded at a density of 1.0 × 10^5^ cells per well onto 12-well culture dishes. After 24 h, the medium was changed with an adipogenic induction medium (IM) containing minimum essential medium-alpha (*α*-MEM; Hyclone Laboratories Inc.), 10% FBS, 1% PS, 1 *μ*M dexamethasone (Sigma Aldrich, St. Louis, MO, USA), 0.5 mM 3-isobutyl-1-methylxanthine (Cayman Chemical, Ann Arbor, ML, USA), 100 *μ*M indomethacin (Sigma Aldrich), 10 mg/mL insulin (cell application, San Diego, CA, USA), and each of the three extracts at various concentrations. After four days, the medium was replaced with an adipogenic maintenance medium (MM) containing *α*-MEM, 10% FBS, 1% PS, 10 mg/mL insulin, and each extract. The medium was replaced every two days during the adipogenic differentiation.

### 2.4. Cytotoxicity Assay

3T3-L1 cells were seeded at a density of 1.0 × 10^3^ cells per well in 96-well plates and incubated overnight at 37°C. Then, the three types of *L. plantarum* extracts were treated at various concentrations (25, 50, 100, and 150 *μ*g/mL). The cytotoxicity of the extracts was evaluated with a lactate dehydrogenase (LDH) cytotoxicity detection kit (Takara, Tokyo, Japan) *in vitro*. To detect the maximum LDH enzyme activity that can be released from the cells, the medium containing 1% Triton X-100 was used as a positive control. After following a detailed product manual, the optical density was measured by an Emax Plus Microplate Reader (Molecular Devices, Sunnyvale, CA, USA) at 492 nm wavelength. To obtain the cytotoxicity (%), the following equation was used.

(1) $$\begin{array}{c} \text{Cytotoxicity}\left(\%\right)=\frac{\left(\text{A}-\text{negative control}\right)}{\left(\text{positive control}-\text{negative control}\right)}\times 100 \end{array}$$

A, (mixture of the extract and the medium in which cells were cultured)-(background). Background, mixture of the extract and the medium with no cell.

### 2.5. Cell Viability Assay

3T3-L1 cells were seeded at a density of 1.0 × 10^3^ cells per well in 96-well plates and incubated at 37°C in 5% CO_2_ with humidity. After 24 h, the culture medium was replaced with the medium containing the respective extracts at various concentrations, i.e., 50, 100, and 150 *μ*g/mL. Then, cell viability was measured by an EZ-Cytox kit (Daeil Lab Service, Seoul, Korea), using the water-soluble tetrazolium salt (WST) method. After 30 min incubation at 37°C, the absorbance was measured at 450 nm with the microplate reader.

### 2.6. Oil Red O Staining and Triglyceride (TAG) Assay

To compare the lipid accumulations, Oil Red O staining was performed. After eight days, the fully differentiated 3T3-L1 cells were washed with PBS, fixed with 4% formaldehyde, and stained with the Oil Red O solution (Sigma Aldrich) for 30 min. The stained adipocytes were observed by an EVOS CL Core microscope (Life Technologies). To compare the relative lipid accumulation, the Oil Red O in differentiated adipocytes was dissolved in isopropyl alcohol (Sigma Aldrich) and quantified by measuring the absorbance at 500 nm using the microplate reader. The results were analyzed as a percentage of the control, which was considered to be 100%. The formula to present the relative lipid accumulation was (*A*_sample_ − *A*_blank_)/(*A*_control_ − *A*_blank_) × 100.

To measure the TAG concentration in the differentiated adipocytes for eight days, the cells were washed with PBS and detached by using a rubber policeman. Subsequently, a triglyceride colorimetric assay kit (Cayman Chemical) was followed according to the instruction manual.

### 2.7. Gene Expression Analysis with Real-Time PCR

3T3-L1 preadipocytes were cultured in the adipogenic IM and MM with each extract for eight days, and the total mRNA was isolated from the differentiated adipocytes using a Pure Link™ RNA mini kit (Life Technologies). Then, cDNA was synthesized by M-MLV reverse transcriptase (Promega Corporation, Fitchburg, MA, USA) according to the manufacturer's instructions. Real-time PCR was conducted using a SYBR Premix Ex Taq II (Takara) and 7500 Real-Time PCR System (Applied Biosystems Inc., Carlsbad, CA, USA). The specific primer sequences to amplify adipogenesis-related marker genes are listed in Table 1. The PCR reaction was performed for 30 s at 95°C, followed by 40 amplification cycles of 5 s at 95°C and 34 s at 60°C. The comparative C_T_ method was used to measure the level of expression. *Glyceraldehyde 3-phosphate dehydrogenase* (*Gapdh*) was used as a housekeeping gene for normalization.

### 2.8. Western Blot Analysis

The proteins were extracted from the fully differentiated 3T3-L1 cells using passive lysis buffer (Promega Corporation, Fitchburg, WI, USA) supplemented with a proteinase inhibitor (MedChemExpress, Monmouth Junction, NJ, USA) and phosphatase inhibitor (MedChemExpress), referring to the manufacturer's descriptions, and were quantified by BCA assay kit (Life Technologies). The proteins were separated by sodium dodecyl sulfate-polyacrylamide gel electrophoresis and immunoblotted with the indicated antibodies: peroxisome proliferator-activated receptor gamma (PPAR*γ*; dilution 1 : 1000; Cell Signaling Technology, Beverly, MA, USA), CCAAT/enhancer-binding protein alpha (C/EBP*α*; dilution 1 : 1000; Cell Signaling Technology), fatty acid-binding protein 4 (FABP4; dilution 1 : 1000; Cell Signaling Technology), fatty acid synthase (FAS; dilution 1 : 1000; Cell Signaling Technology), protein kinase B (AKT; dilution 1 : 1000; Bioss, Woburn, MA, USA), phospho-Akt (p-AKT; dilution 1 : 2000; Cell Signaling Technology), tumor necrosis factor-alpha (TNF-*α*; dilution 1 : 1000; Cusabio Life Science, Wuhan, China), and GAPDH (dilution 1 : 2000; BioLegend, San Diego, CA, USA). GAPDH was used to achieve equal loading of protein. The protein signals on the membranes were developed using ECL western blotting substrate (Daeil Lab Service).

### 2.9. Glucose Uptake Assay

To determine the level of glucose uptake in mature adipocytes, a fluorescent derivative of glucose (2-NBDG; Cayman) was used. First, 3T3-L1 preadipocytes were differentiated in the IM and MM with each extract. After eight days of incubation, fully differentiated adipocytes were washed with PBS and seeded into 96-well plates using low-glucose DMEM (WELGENE) supplemented with 10% FBS and incubated for one day. Then, the positive control was initially treated with 1 *μ*M insulin for 30 min, and all cells were treated with or without 2-NBDG. After incubation for 4 h at 37°C, the cells were washed with cold PBS, and fluorescence measurements were obtained using a fluorescence microplate reader (SpectraMax ABS Plus; Molecular Devices) at ex/em = 450/535 nm. To capture fluorescence images, 3T3-L1 adipocytes were cultured on sterilized glass coverslips in low-glucose DMEM containing 10% FBS, and the glucose uptake assay was performed as described above. After washing with cold PBS, the cells were mounted on slides using a mounting solution with 4′,6-diamidino-2-phenylindole (Life Technologies) fluorescent stain. All slides were observed with a LSM 800 (Zeiss, Baden-Württemberg, Germany).

### 2.10. Statistical Analysis

Results are presented as the mean ± standard deviation. Statistical analysis was determined using a one-way analysis of variance (ANOVA), followed by Tukey's post hoc test. For statistical significance, GraphPad Prism V5.0 software (GraphPad Software, La Jolla, CA, USA) was used, and a significance value was marked as ^∗^*p* < 0.05, ^∗∗^*p* < 0.01, and ^∗∗∗^*p* < 0.001.

## 3. Results

### 3.1. The Identification of LS, LP, and L14 as *L. plantarum 1* by the API 50 CHL Test

**Figure 1:**
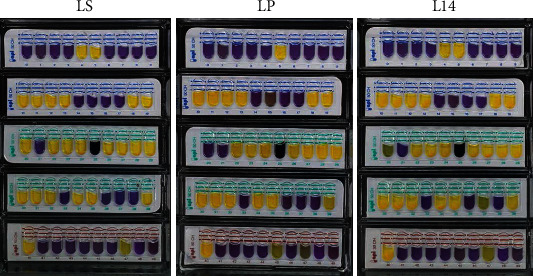
Identification of LS, LP, and L14 in *Lactobacillus plantarum* (*L. plantarum*) via the API 50 CHL test. API 50 CHL strip identification panels, inoculated with LS, LP, and L14, respectively. Images present all three lactic acid bacteria identified as *L. plantarum 1* with high accuracy. Purple: fermented carbohydrate; yellow and black (test number 25): nonfermented carbohydrate.

LS, LP, and L14 inoculated into the API 50 CHL medium were placed onto API CHL 50 strips and incubated for 48 h at 37°C. Carbohydrate fermentation patterns on API 50 CHL test are shown in Figure 1 and summarized in Table 2. LS, LP, and L14 were identified as *L. plantarum* 1, showing 99.9%, 99.9%, and 99.5% identity, respectively (Table 2). However, the utilization of several carbohydrates was different based on the carbohydrate test number 4, 15, 20, 37, 47, and 49 (Table 2). The comparison of the carbohydrate fermentation patterns of the three LABs suggests that distinct metabolic abilities are found among the different strains, despite being the same species.

### 3.2. Cell Viability and Cytotoxicity of LS, LP, and L14 Extract Treatments in 3T3-L1 Cells

**Figure 2:**
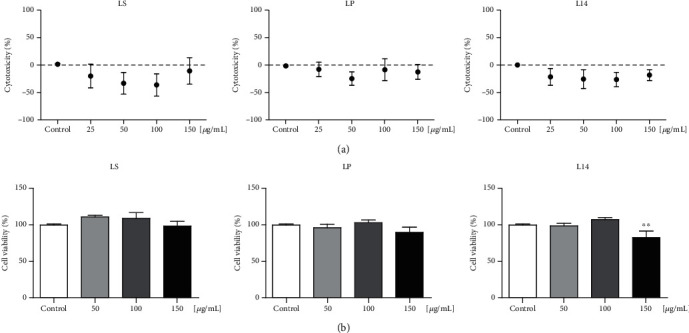
Cell viability and cytotoxicity of LS, LP, and L14 extracts on 3T3-L1 cells. (a) A lactate dehydrogenase cytotoxicity assay was used to detect the cytotoxicity upon exposure to four concentrations of three *L. plantarum* extracts (*n* = 3). (b) Water-soluble tetrazolium salt assay was performed to assess the effects of *L. plantarum* extracts on the viability (*n* = 3). The results showed that 50 and 100 *μ*g/mL concentrations of three *L. plantarum* extracts were nontoxic to cell growth and maintenance during adipogenic differentiation. Error bars represent the standard deviation of the mean. ^∗∗^*p* < 0.01, one-way ANOVA followed by Tukey's post hoc test was used.

The LDH cytotoxicity assay was used to investigate whether the three extracts were toxic to 3T3-L1 cells. As shown in Figure 2(a), the three extracts exhibited noncytotoxicity in 3T3-L1 preadipocytes up to the concentration of 150 *μ*g/mL. Next, the WST assay was performed to evaluate the effect of each *L. plantarum* extract on the cell viability during adipogenic differentiation. The cell viability was reduced by treatment with 150 *μ*g/mL of the L14 extract (Figure 2(b)). These results suggest that concentrations of 50 and 100 *μ*g/mL of all *L. plantarum* extracts are nontoxic to cells, and so these concentrations were used in subsequent experiments.

### 3.3. Inhibitory Effect on the Lipid Accumulation of LS, LP, and L14 Extracts

**Figure 3:**
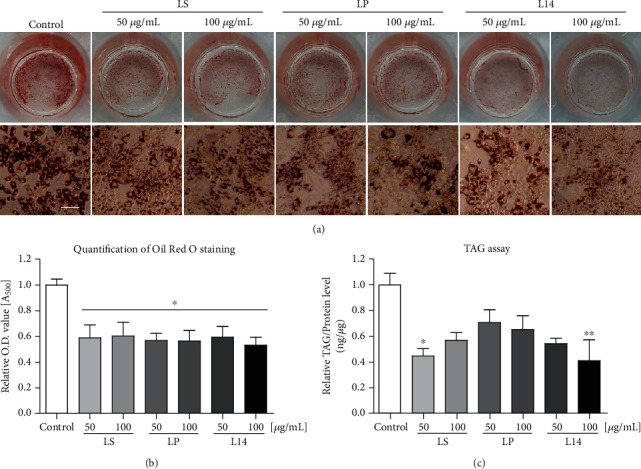
Effects of LS, LP, and L14 extracts on lipid accumulation in 3T3-L1 adipocytes. (a) Images of Oil Red O staining of fully differentiated 3T3-L1 cells with two concentrations of each of the three *L. plantarum* extracts. (b) Quantification of Oil Red O staining demonstrated that lipid droplet accumulation was inhibited in all extracts groups (*n* = 3). (c) Triglyceride (TAG) assay indicated that significantly greater inhibition of TAG accumulation was seen in the 100 *μ*g/mL L14-extract-treatment group (*n* = 3). Error bars represent the standard deviation of the mean. ^∗^*p* < 0.05, ^∗∗^*p* < 0.01, one-way ANOVA followed by Tukey's post hoc test was used. Scale bar = 100 *μ*m.

To determine whether the three *L. plantarum* extracts inhibited adipogenic differentiation of mouse preadipocytes, Oil Red O staining and TAG assay were performed. As shown in Figure 3(a), the lipid droplets detected by the Oil Red O solution were decreased in all extracts-treated groups. The relative lipid contents were quantified, as shown in Figure 3(b), which represents Figure 3(a). The TAG contents of differentiated 3T3-L1 cells were most repressed by 100 *μ*g/mL of the L14 extract (Figure 3(c)). This indicates that the L14 extract inhibits lipid accumulation the most in the differentiated 3T3-L1 adipocytes.

### 3.4. Changes in the Expression Levels of Adipogenesis-Related Factors by LS, LP, and L14 Extracts

**Figure 4:**
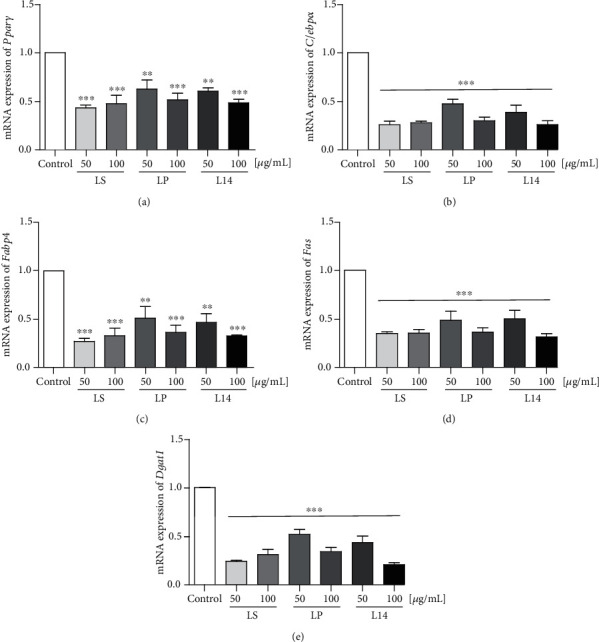
Effects of LS, LP, and L14 extracts on the gene expression of adipogenesis-related factors in differentiated 3T3-L1 cells. The expression of adipogenesis-related genes ((a) *Pparγ*, (b) *C/ebpα*, (c) *Fabp4*, (d) *Fas*, and (e) *Dgat1*) were analyzed by real-time PCR (*n* = 3). The results show that all expressions of adipogenesis-related factors were downregulated by the three extracts. *Gapdh* was used for normalization. Error bars represent the standard deviation of the mean. ^∗∗^*p* < 0.01, ^∗∗∗^*p* < 0.001, one-way ANOVA followed by Tukey's post hoc test was used.

**Figure 5:**
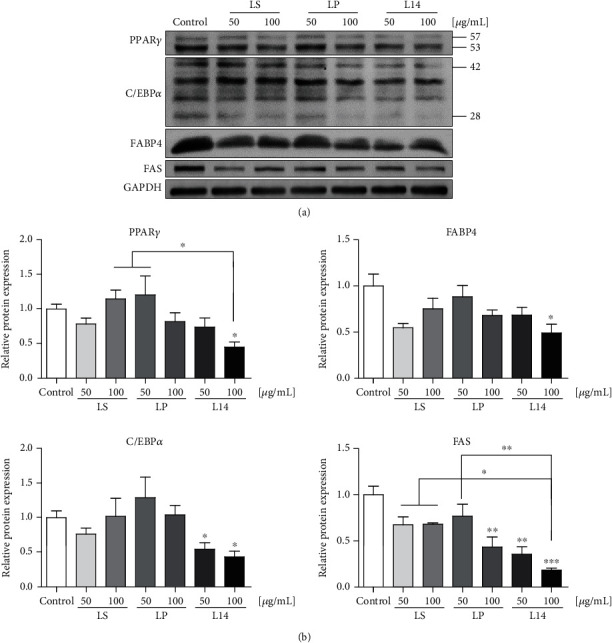
Effects of LS, LP, and L14 extracts on adipogenesis-related protein expressions in 3T3-L1 adipocytes. (a) The protein levels of PPAR*γ*, C/EBP*α*, FABP4, and FAS were analyzed by western blotting (*n* = 3). (b) The intensity of the protein band was quantified, relative to GAPDH. As a result, the L14 extract most significantly inhibited the protein expressions of adipogenesis-related factors. Error bars represent the standard deviation of the mean. ^∗^*p* < 0.05, ^∗∗^*p* < 0.01, ^∗∗∗^*p* < 0.001, one-way ANOVA followed by Tukey's post hoc test was used.

The expression of adipogenesis-related factors such as PPPAR*γ*, C/EBP*α*, FABP4, FAS, and DGAT1 was evaluated to confirm and compare the inhibitory effects of the *L. plantarum* extracts on adipogenic differentiation (Figures 4 and 5). The expression levels of all adipogenesis-related genes were significantly downregulated in all extract-treated groups and were dose-dependently inhibited by the LP and L14 extracts (Figure 4). The protein expression of adipogenesis-related factors tended to decrease in a concentration-dependent manner by the LP and L14 extract treatments (Figure 5(b)). Whereas the mRNA expression of the adipogenesis-related genes was significantly decreased in the LS-extract-treatment groups (Figure 4), there was no significant decrease in any protein level (Figure 5(b)). Interestingly, all adipogenesis-related factor gene and protein levels were remarkably decreased by 100 *μ*g/mL of the L14 extract (Figures 4 and 5(b)). Moreover, the expression of PPAR*γ* and FAS proteins was significantly decreased in the L14-extract-treatment groups than in the LP- and LS-extract-treatment groups (Figure 5(b)). Taken together, among the three extracts, the L14 extract has the best effect on adipogenic differentiation reduction.

### 3.5. Comparison of the Stimulated Effect of Glucose Uptake into 3T3-L1 Cells and Changes in the Expression of Insulin Resistance-Related Proteins Regulated by LS, LP, and L14 Extracts

**Figure 6:**
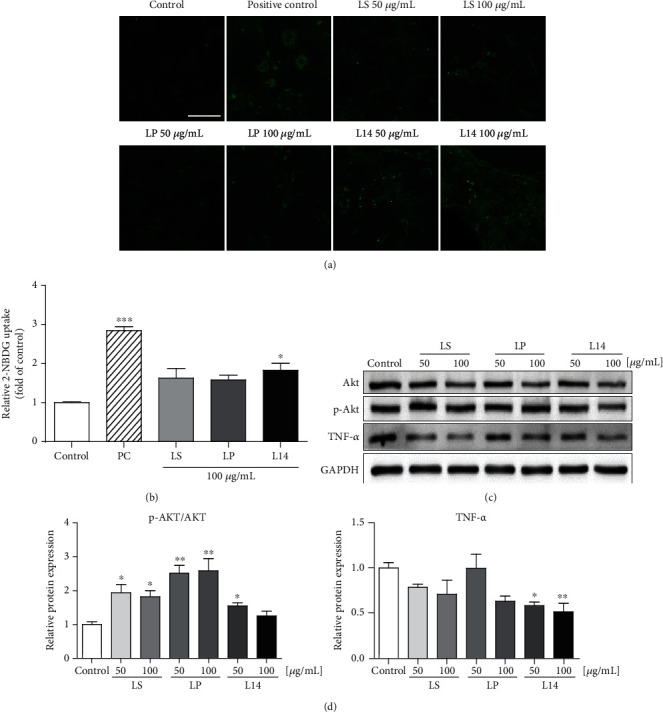
Effects of LS, LP, and L14 extracts on the amount of glucose uptake into 3T3-L1 adipocytes and insulin resistance-related protein expressions. (a) 2-NBDG, a fluorescent-tagged glucose analog, was used to evaluate the efficacy of transport to adipocytes. The treatment of 1 *μ*M insulin was used as a positive control. (b) The amount of the glucose uptake by three extracts at a concentration of 100 *μ*g/mL was detected using the fluorescence microplate reader (*n* = 3). (c) Western blot for insulin resistance-related proteins (TNF-*α*, AKT, and p-AKT) levels (*n* = 3). (d) Quantitative analysis of p-AKT and TNF-*α* expression levels in (c). GAPDH was used for normalization. Error bars represent the standard deviation of the mean. ^∗^*p* < 0.05, ^∗∗^*p* < 0.01, ^∗∗∗^*p* < 0.001, one-way ANOVA followed by Tukey's post hoc test was used. Scale bar = 100 *μ*m. PC: positive control.

To determine whether *L. plantarum* extracts affected the obesity-induced insulin resistance, 2-NBDG uptake was evaluated in fully differentiated adipocytes at all extract 50 and 100 *μ*g/mL concentrations. The green fluorescence intensity of 2-NBDG was measured using fluorescence microscopy to assess the transport efficacy of 2-NBDG into 3T3-L1 cells (Figure 6(a)). The images show that the intensities of the fluorescence were enhanced in a concentration-dependent manner (Figure 6(a)). Additionally, 2-NBDG uptake was detected with the fluorescence microplate reader after 100 *μ*g/mL treatment of each of the three extracts (Figure 6(b)). In comparing the signal intensities, 100 *μ*g/mL L14-extract-treated group significantly exhibited stronger activities, whereas LP- and LS-extract-treated groups showed weak activities (Figure 6(b)). These results suggest that the L14 extract stimulated insulin-induced glucose uptake the most and could regulate obesity-induced glucose metabolic disorders.

The expressions of TNF-*α* and AKT, which were well known to be insulin resistance-related factors in adipocytes, were evaluated to observe the mechanistic effect for the insulin sensitivity by *L. plantarum* extracts (Figure 6(c)). Quantification of the protein expressions was presented by bar graph in Figure 6(d). The results showed that the protein expressions of TNF-*α* were most reduced by the L14 extract (Figures 6(c) and 6(d)). Quantitative analysis of p-AKT expression levels was promoted in 50 *μ*g/mL of the L14 extract, as well as all concentrations of the LP and LS extract (Figure 6(d)).

## 4. Discussion

In 1907, Metchnikoff and Tissier's researches suggested the first scientific use of probiotics in bacteria [28, 29]. Since then, significant advances have been made in the extraction and screening, identification, cultivation, and the demonstration of beneficial effects on the human body, including the benefits of probiotics against obesity [11–14, 30]. Many bacteria are still being discovered and reported for species- and strain-specific effects and different metabolic abilities [31–34]. Furthermore, there are differences in the advantageous effects of bacterial species and strains that may be understood through screening and comparative analyses [35–37]. Recently, numerous bacterial strains have been screened and identified as probiotics that ameliorate metabolic disorders in obese mice [36]. However, the antiobesity and antidiabetic effects of bacterial extracts are rarely evaluated, and comparative analyses do not compare the effectiveness of each extract. Thus, this study is aimed at comparing the beneficial effects of newly identified *L. plantarum* extracts in treating obesity and insulin resistance.

Through API 50 CHL test, LS, LP, and L14 which were isolated from different products were identified as *L. plantarum* 1 (Figure 1). The API 50 CHL system can be useful for characterizing the bacteria below species level, but it has limitations that cannot identify all unknown bacteria [38]. For this reason, 16S rRNA sequencing was supplementally performed to clarify that the three LABs were *L. plantarum* (data not shown). The comparison of the carbohydrate fermentation patterns of the three LABs suggests that distinct metabolic abilities are found among the different strains, despite being the same species (Table 2). In previous studies, the carbohydrate preference varied among *Bifidobacterium* species and strains [39, 40]. Additionally, bacteria produce completely different metabolites depending on what they consume, and these metabolites play a beneficial role in humans [32]. A recent metabolomics study demonstrates that the regulation of the host's physiology is associated with specific bacterial metabolites [41]. Considering that LS, LP, and L14 consume different carbohydrates, it can be assumed that they will cause different antiobesity effects.

Oil red O staining and TAG assay were used to measure the reduced lipid contents by the *L. plantarum* extracts (Figure 3). Figures 3(a) and 3(b) showed that the lipid droplet contents were decreased by all extracts treatment. Unlike the principal of Oil Red O staining that is used to detect the lipid droplets containing neutral lipids such as triacylglycerol and sterol esters [42], the TAG assay was measured predominantly intracellular TAG contents was based on protein contents. For this reason, the results of Oil Red O staining and TAG assay were slightly different tendency. As shown in Figure 3(c), the TAG contents were most repressed by 100 *μ*g/mL of the L14 extract, which indicates the highest inhibitory ability to lipid accumulation.

In this study, the expression of adipogenesis-related factors such as PPPAR*γ*, C/EBP*α*, FABP4, FAS, and DGAT1 was evaluated to confirm the inhibitory effect of the *L. plantarum* extracts on adipogenic differentiation (Figures 4 and 5). It is well known that PPAR*γ* activates enzymes that promote TAG synthesis in adipocytes; namely, its activity is directly related to fat content [43]. In this respect, the protein expression of PPAR*γ* was closely analogous to the result of the TAG assay, which indicates that the adipose content was lowest in the 100 *μ*g/mL L14-extract-treated 3T3-L1 cells (Figures 3(c) and 5). PPAR*γ* and C/EBP*α* are early regulators of adipogenic induction [44], and they produce a positive feedback loop between each other [45]. Hence, the similarity in the protein expressions of C/EBP*α* and PPAR*γ* indicates that they cooperated in the preadipocytes, and their lowest expression revealed that the differentiation was most suppressed by the mature adipocyte-treated with 100 *μ*g/mL of the L14 extract (Figure 5). Other factors responsible for lipid accumulation and adipocyte maturation, FABP4 and FAS [46], were also most downregulated by 100 *μ*g/mL of the L14 extract (Figure 5). Interestingly, in a previous study, a deficiency of FABP or DGAT1 caused protective effects in obesity and insulin resistance [47, 48]. As shown in Figures 4(c) and 5(b), 100 *μ*g/mL of the L14 extract treatment showed protection from insulin resistance through the inhibition of the mRNA and protein expressions of FABP4 and the mRNA level of *Dgat1* (Figures 4(c) and 5). In summary, these results suggest that the L14 extract could be a feasible therapeutic strategy for obesity and type 2 diabetes.

To confirm the influence of the *L. plantarum* extracts on obesity-induced insulin resistance, the glucose uptake assay and the western blotting for insulin resistance-related factors were performed (Figure 6). The green fluorescence intensity of 2-NBDG was strong with all concentrations of L14 extract (Figures 6(a) and 6(b)). Adipose tissue has been reported to synthesize numerous cytokines and growth factors [49]. One of the signaling molecules that are produced by adipose tissue is TNF-*α*, which has been proven to regulate almost every aspect of adipose biological processes as well as metabolic diseases [50]. Increased TNF-*α* concentrations might induce insulin resistance via changes in the adipocyte insulin signaling pathway, as suggested in one study [49]. Hence, TNF-*α* is well known to be an important mediator of insulin resistance in adipose tissue through insulin-receptor signaling in rodents and humans [49–54]. In particular, an increased concentration of TNF-*α* in insulin-resistant fat cells decreases the insulin signaling cascade, including the phosphorylation of AKT proteins [49, 55, 56]. In this study, TNF-*α* expression and phosphorylation of AKT were analyzed, and the protein expression levels of TNF-*α* were reduced by the L14 extracts (Figures 6(c) and 6(d)). However, the p-AKT expression levels were promoted by 50 *μ*g/mL of the L14 extract, as well as by all concentrations of the LP and LS extracts (Figure 6(d)). This indicates that the overall increased expression of p-AKT might be limited in representing all insulin signaling due to another role of AKT in adipogenic differentiation. Taken together, the reduced protein levels of TNF-*α* by *L. plantarum* extracts remarkably stimulated glucose uptake in differentiated adipocytes and induced insulin sensitivity compared with that of the control group. The L14 extract significantly inhibited obesity-induced insulin resistance and adipogenic differentiation.

Obesity has been reported to cause the development of vascular and metabolic diseases, such as dyslipidemia and cardiovascular disease, through the development of insulin resistance [6]. Additionally, it has been reported that obesity and insulin resistance have existed for years before the appearance of the abovementioned abnormalities [6, 57]. Hence, this study suggests that the beneficial effects of the L14 extract demonstrate its potential as a health product for preventing type 2 diabetes and cardiovascular disease. In previous research, antidiabetic drugs such as thiazolidinedione improved insulin sensitivity, but they had reported side effect of weight gain [58]. Thus, new safer antidiabetic agents with antiobesity properties and minimal side effects are needed for developing of antidiabetic therapeutics. In this regard, the L14 extract might have therapeutic potential for type 2 diabetes by activating the insulin signaling pathways following TNF-*α* reduction. However, the exact components of the extracts and molecular mechanisms of 3T3-L1 cells are yet to be explained. Further research is required to identify functional molecules in the extracts that exhibit antiobesity and antidiabetic effects with a specific molecule that can demonstrate the differences in effects in a comparison of the compositions of each extract. Recently, comparisons and analyses of the mechanisms of the probiotic function of individual strains were studied [59], and an animal research provided clues on how to use probiotics more effectively for insulin resistance through a comparison of the strain-specific effects of probiotics on insulin resistance [60]. As strain specificity presents within the same species, our study comparing the beneficial effects of *L. plantarum* extracts may be helpful in the selection of suitable strains for therapeutics or health products. Further studies should be conducted toward developing standardized evaluation methods to facilitate comparisons recommended by our study.

## 5. Conclusions

This study demonstrated the beneficial effects of three newly identified *L. plantarum* extracts related to obesity and insulin resistance. Our findings showed that there are differences in the antiobesity and antidiabetic effects among the three strains in terms of lipid accumulation, adipogenesis-related factors and insulin resistance-related factors, and the glucose uptake into differentiated 3T3-L1 adipocytes. The results also demonstrated that the L14 and LS extracts are more beneficial in decreasing adipogenic differentiation than is the LP extract. The L14 extract is the most salutary for obesity and insulin sensitivity and has a greater ability to attenuate adipogenic differentiation and obesity-induced insulin resistance among the three new *L. plantarum* extracts. Even though the three *L. plantarum* strains are the same species, the degree of their effects may be different. In particular, the L14 extract has the most potential as a therapeutic drug or health supplement product for the treatment of obesity and diabetes; an *in vivo* study is necessary to validate the effects reported in this study.

## Figures and Tables

**Table 1:** Primer sequences used for real-time PCR.

Gene		Primer sequence
Mouse genes		
*Pparγ*	Forward	5′-TTCAGAAGTGCCTTGCTGTG-3′
	Reverse	5′-GCTGGTCGATATCACTGGAGA-3′

*C/ebpα*	Forward	5′-GGTGCGTCTAAGATGAGGGA-3′
	Reverse	5′-CCCCCTACTCGGTAGGAAAA-3′

*Fabp4*	Forward	5′-AAGGTGAAGAGCATCATAACCCT-3′
	Reverse	5′-TCACGCCTTTCATAACACATTCC-3′

*Fas*	Forward	5′-ATCCGGAACGAGAACACGATCT-3′
	Reverse	5′-AGAGACGTGTCACTCCTGGACTT-3′

*Dgat1*	Forward	5′-ACCGCGAGTTCTACA-3′
	Reverse	5′-AGGGGAACGCTCACTAGGTA-3′

*Gapdh*	Forward	5′-AGGTCGGTGTGAACGGATTTG-3′
	Reverse	5′-TGTAGACCATGTAGTTGAGGTCA-3′

**Table 2:** API 50 CHL test and probable identification of the three newly identified *Lactobacillus* isolates.

Test number	0	1	2	3	4	5	6	7	8	9	10	11	12	13	14	15	16	17	18	19	20	21	22	23	24	25	26	27
Strains code	Control	Glycerol	Erythritol	D-arabinose	L-arabinose	Ribose	D-xylose	L-xylose	Adonitol D-adonitol	Methyl-DB-xylopyranosicle	D-galactose	D-glucose	D-fructose	D-mannosee	L-sorbose	Rhamnose	Dulcitol	Inositol	Mannitol	Sorbitol	*α*-methyl-D-annoside	*α*-methyl-D-glucoside	N-acetyl-glucosamine	Amygdalin	Arbutin	Esculin	Salicin	Cellobiose
LS	**—**	**±**	**—**	**—**	**+**	**+**	**—**	**—**	**—**	**—**	**+**	**+**	**+**	**+**	**—**	**—**	**—**	**—**	**+**	**+**	**+**	**—**	**+**	**+**	**+**	**+**	**+**	**+**
LP	**—**	**±**	**—**	**—**	**—**	**+**	**—**	**—**	**—**	**—**	**+**	**+**	**+**	**+**	**—**	**±**	**—**	**—**	**+**	**+**	**—**	**—**	**+**	**+**	**+**	**+**	**+**	**+**
L14	**—**	**±**	**—**	**—**	**+**	**+**	**—**	**—**	**—**	**—**	**+**	**+**	**+**	**+**	**—**	**±**	**—**	**—**	**+**	**+**	**±**	**—**	**+**	**+**	**+**	**+**	**+**	**+**
Test number	28	29	30	31	32	33	34	35	36	37	38	39	40	41	42	43	44	45	46	47	48	49						
Strains code	Maltose	Lactose	Melibiose	Sucrose	Trehalose	Inulin	Melezitose	Raffinose	Starch	Glycogen	Xylitol	Gentiobiose	D-turanose	D-lyxose	D-tagatose	D-fucose	L-fucose	D-arabitol	L-arabitol	Gluconate	2-keto-Gluconate	5-keto-Gluconate	Identification			Accuracy (%)		
LS	**+**	**+**	**+**	**+**	**+**	**—**	**+**	**+**	**—**	**—**	**—**	**+**	**+**	**—**	**—**	**—**	**—**	**±**	**—**	**+**	**—**	**±**	*Lactobacillus plantarum 1*			99.9		
LP	**+**	**+**	**+**	**+**	**+**	**—**	**+**	**+**	**—**	**—**	**—**	**+**	**+**	**—**	**—**	**—**	**—**	**±**	**—**	**±**	**—**	**±**				99.9		
L14	**+**	**+**	**+**	**+**	**+**	**—**	**+**	**+**	**—**	**±**	**—**	**+**	**+**	**—**	**—**	**—**	**—**	**±**	**—**	**+**	**—**	**—**				99.5		

+: fermented carbohydrate; −: nonfermented carbohydrate; ±: variable.

## Data Availability

The data used to support the findings of this study are available from the corresponding author upon request.
